# Dose Response of Bumetanide on Aquaporins and Angiogenesis Biomarkers in Human Retinal Endothelial Cells Exposed to Intermittent Hypoxia

**DOI:** 10.3390/ph14100967

**Published:** 2021-09-24

**Authors:** Sibel Guzel, Charles L. Cai, Jacob V. Aranda, Kay D. Beharry

**Affiliations:** 1 Department of Pediatrics, Division of Neonatal-Perinatal Medicine, State University of New York, Downstate Medical Center, Brooklyn, NY 11203, USA; sibelgzel@yahoo.com (S.G.); Charles.cai@downstate.edu (C.L.C.); Jacob.aranda@downstate.edu (J.V.A.); 2 Department of Ophthalmology, State University of New York, Downstate Medical Center, Brooklyn, NY 11203, USA; 3 State University of New York Eye Institute, Brooklyn, NY 11203, USA

**Keywords:** angiogenesis, aquaporins, bumetanide, human microvascular retinal endothelial cells, intermittent hypoxia, vascular endothelial growth factor

## Abstract

Aquaporins (AQPs) are important for regulating cellular water, solute transport, and balance. Recently, AQPs have also been recognized as playing a key role in cell migration and angiogenesis. In the retina, hypoxia induces vascular endothelial growth factor (VEGF), a potent angiogenic and vascular permeability factor, resulting in retinal edema, which is facilitated by AQPs. Bumetanide is a diuretic agent and AQP 1–4 blocker. We tested the hypothesis that bumetanide suppression of AQPs ameliorates intermittent hypoxia (IH)-induced angiogenesis and oxidative stress in human microvascular retinal endothelial cells (HMRECs). HMRECs were treated with a low-dose (0.05 µg/mL) or high-dose (0.2 µg/mL) of bumetanide and were exposed to normoxia (Nx), hyperoxia (50% O_2_), or IH (50% O_2_ with brief hypoxia 5% O_2_) for 24, 48, and 72 h. Angiogenesis and oxidative stress biomarkers were determined in the culture media, and the cells were assessed for tube formation capacity and AQP-1 and -4 expression. Both doses of bumetanide significantly decreased oxidative stress and angiogenesis biomarkers. This response was reflected by reductions in tube formation capacity and AQP expression. These findings confirm the role of AQPs in retinal angiogenesis. Therapeutic targeting of AQPs with bumetanide may be advantageous for IH-induced aberrant retinal development.

## 1. Introduction

Retinopathy of Prematurity (ROP) has become the leading cause of preventable childhood blindness throughout the world, as the quality of neonatal care has improved the survival of very preterm infants [1]. Out of the more than 1.5 million children who are blind worldwide, approximately 50,000 children are reported to be blind due to ROP [2]. ROP only affects 4000 to 16,000 premature infants in the United States [3]. Decades of research dedicated to maximizing the survival and the minimizing complications of ROP have helped optimize the management of oxygen supplementation for premature infants [2]. ROP is an oxygen-induced disease and develops in two phases [4,5]. In the first phase, the relative hyperoxia of the extrauterine environment results in vasoconstriction and the cessation of normal vascular development, and the second phase occurs when a pathologic compensatory mechanism with aberrant neovascularization ensues. If the second phase leads to a significant amount of fibrovascular proliferation and is left untreated, then exudative, tractional, or combined type retinal detachment can occur [2]. Such vascular development is a product of astrocyte interactions and trophic factor gradients established by glia and retinal ganglion cells [6].

Vascular endothelial growth factor (VEGF) has been shown to play an important role in normal angiogenesis and is upregulated in the second phase of ROP development, causing neovascularization. Severe ROP can have a negative impact on long-term visual and neurodevelopmental outcomes in extremely premature infants. The treatment of ROP maximizes visual outcomes through preventing retinal detachment and through preserving the area of viable retina [7]. Currently, approved treatments for severe ROP are cryotherapy or laser photocoagulation of the avascular regions of retina, but the use of pharmacologic interventions, such as anti-VEGF therapies, have significantly increased [7]. There are significant side effects from both laser surgery and/or anti-VEGF therapy treatments [8]. Laser therapy is often painful, and tissue destruction places a limit on further treatments [9,10]. Current anti-VEGF drugs, injected intravitreally to bypass the anterior and posterior access barriers, have serious associated risks, such as retinal tear/detachment [11,12], endophthalmitis, cataracts, vitreous and retinal hemorrhage, choroidal rupture, leakage into the fellow eye, and increased risk of neurological disabilities [13,14,15,16]. In addition to the general complications of intravitreal drug delivery (endophthalmitis, retinal detachment, traumatic cataract, subconjunctival hemorrhage, uveitis, and pain), possible systemic complications of anti-VEGF agents have also been cited as a concern [17,18,19,20,21,22,23,24,25]. The development of new treatment strategies for pathological neovascularization is warranted.

Aquaporins (AQPs) are a family of small integral membrane proteins that facilitate trans-epithelial fluid transport and increase cell membrane permeability by 5–50 times compared to that in membranes, where water primarily moves through the lipid bilayer [26]. AQPs play important roles in physiological functions and human diseases. There are 13 types of AQPs (numbered 0 through 12) that have been identified [27], all of which are present in ocular tissues [28]. However, AQP-1 and AQP-4 have been shown to be the most abundantly expressed AQP in the rodent retina, and both play key roles in retinal development [29]. AQP1 is expressed in the outer retina, mainly in a subpopulation of amacrine cells and in photoreceptor cells [29], and has a polarized distribution in retinal pigment epithelium (RPE), which contributes to efficient transepithelial water transport, retinal attachment maintenance, and subretinal edema prevention. AQP-4 is predominantly expressed in the muller glial cells of the retinal astrocyte foot processes in close contact with endothelial cells (ECs). It has been shown to co-localize and interact with the inwardly rectifying potassium channel Kir4.1, the major channel involved in the spatial buffering of the retinal potassium concentration mediated by Müller cells [30]. Its deletion is protective against ischemic injury of the retina [31].

Bumetanide, a loop diuretic and selective AQP-1 as well as -4 inhibitor, is administered to volume-overloaded infants [32] and to neonates with seizures [33]. The anti-seizure effects of bumetanide are mediated by its inhibitory action on the Na^+^K^+^Cl_2_ co-transporter isoform 1 (NKCC1), which transports Cl_2_ into the cell and the KCC2 transporter that moves Cl_2_ out of the cell [34,35]. Simultaneous inhibition of NKCC1 and AQP4 by bumetanide reduces edema and spinal cord tissue destruction in rats [36]. Bumetanide has also been shown to have anti-angiogenesis properties [29,37]. We have previously shown that early postnatal bumetanide reduced the severity of OIR induced by neonatal IH, likely due to the suppression of AQP4 [38]. In the present study, we examined the effects of low and high doses of bumetanide on HMRECs exposed to IH to test the hypothesis that bumetanide reverses intermittent hypoxia (IH)-induced angiogenesis and oxidative stress via AQP suppression.

## 2. Results

### 2.1. Effect of Bumetanide on Oxidative Stress

**Figure 1:**
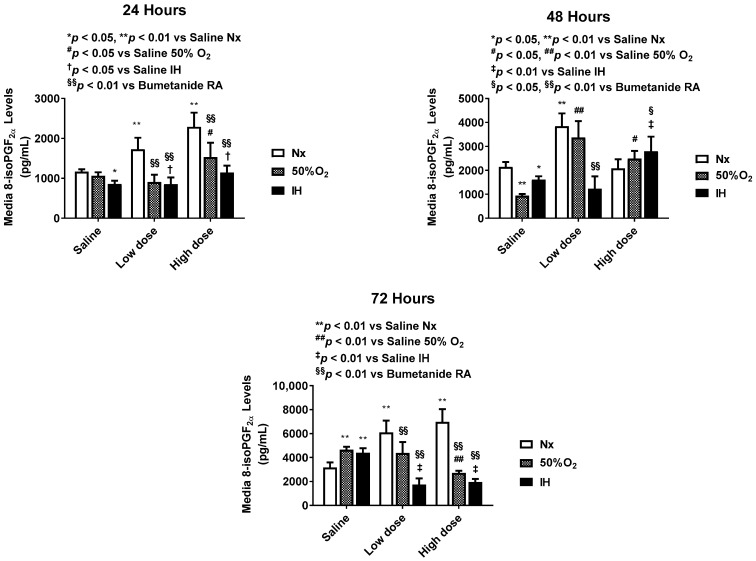
Dose-response effect of bumetanide on 8-isoPGF_2α_ levels in the media from cultured human microvascular retinal endothelial cells (HMRECs) exposed to normoxia (white bar), hyperoxia (50% O_2_, shaded bar), or intermittent hypoxia (IH, black bar) for 24, 48, or 72 h. Data were analyzed using two-way ANOVA with Dunnett’s post hoc test. Data are mean ± SEM (8 samples/group). * *p* < 0.05, ** *p* < 0.01 versus saline RA; ^#^ *p* < 0.05, ^##^ *p* < 0.01 versus saline 50% O_2_; ^†^ *p* < 0.05, ^‡^ *p* < 0.01 vs. saline IH; and ^§^ *p* < 0.05, ^§§^ *p* < 0.01 versus bumetanide treatment in RA.

The biomarker 8-isoPGF_2α_ is a reliable for the determination for oxidative stress. Figure 1 shows the time- and dose-dependent effects of bumetanide on 8-isoPGF_2α_ levels in the media at 24, 48, and 72 h post treatment. Levels of 8-isoPGF_2α_ progressively increased in the placebo saline group exposed to Hx and IH, causing them to peak at 72 h. High-dose bumetanide increased 8-isoPGF_2α_ levels in Nx at 24 and 72 h. However, in Hx and IH, both doses caused an opposite effect.

### 2.2. Effect of Bumetanide on Percentage (%) Cytotoxicity

**Figure 2:**
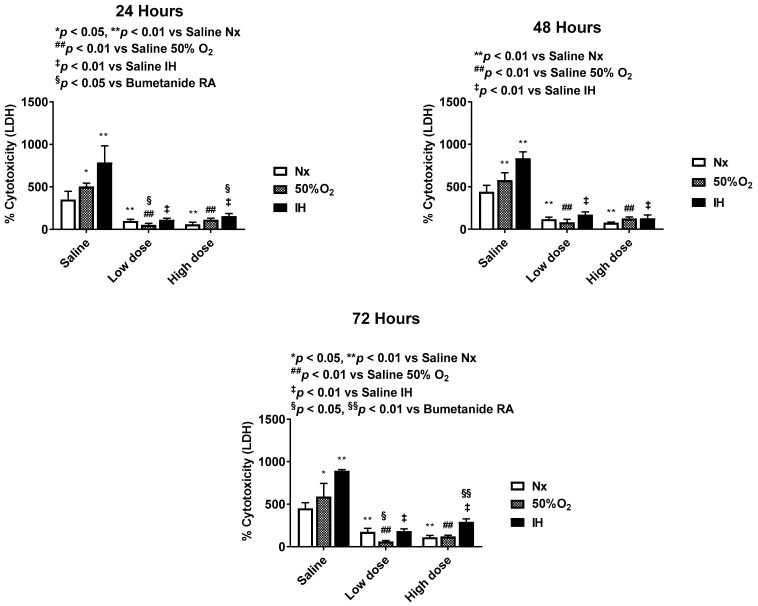
Dose-response effect of bumetanide on % cytotoxicity in the media from cultured human microvascular retinal endothelial cells (HMRECs) exposed to normoxia (white bar), hyperoxia (50% O_2_, shaded bar), or intermittent hypoxia (IH, black bar) for 24, 48, or 72 h. Cytotoxicity was determined using the lactate dehydrogenase (LDH) assay. Data were analyzed using two-way ANOVA with Dunnett’s post hoc test. Data are mean ± SEM (8 samples/group). * *p* < 0.05, ** *p* < 0.01 versus saline RA; ^##^ *p* < 0.01 versus saline 50% O_2_; ^‡^ *p* < 0.01 vs. saline IH; and ^§^ *p* < 0.05, ^§§^ *p* < 0.01 versus bumetanide treatment in RA.

Cell cytotoxicity % is presented in Figure 2. In the placebo saline group, exposure to IH was more toxic to the cells compared to Hx. Both doses of bumetanide significantly suppressed cell cytotoxicity in all oxygen environments. Notably, at 72 h, there was less suppression in IH compared to in RA and Hx.

### 2.3. Effect of Bumetanide on Angiogenesis

**Figure 3:**
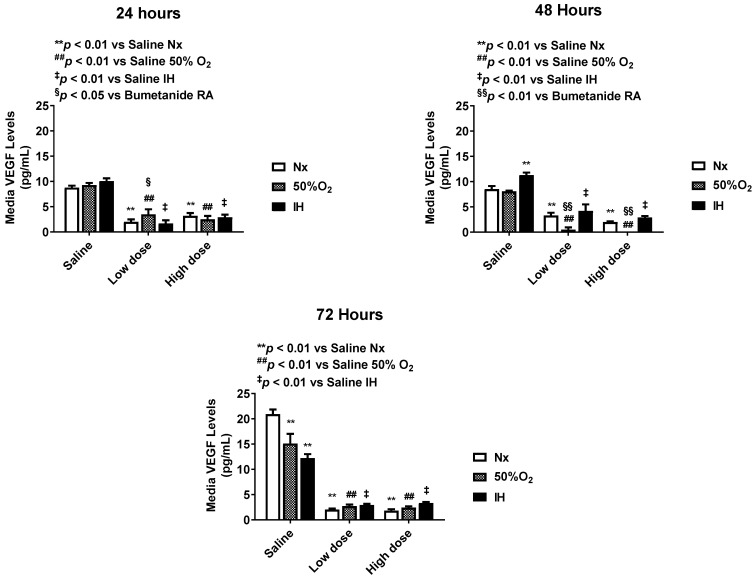
Dose-response effect of bumetanide on VEGF levels in the media from cultured human microvascular retinal endothelial cells (HMRECs) exposed to normoxia (white bar), hyperoxia (50% O_2_, shaded bar), or intermittent hypoxia (IH, black bar) for 24, 48, or 72 h. Data were analyzed using two-way ANOVA with Dunnett’s post hoc test. Data are mean ± SEM (8 samples/group). ** *p* < 0.01 versus saline RA; ^##^ *p* < 0.01 versus saline 50% O_2_; ^‡^ *p* < 0.01 vs. saline IH; and ^§^ *p* < 0.05, ^§§^ *p* < 0.01 versus bumetanide treatment in RA.

**Figure 4:**
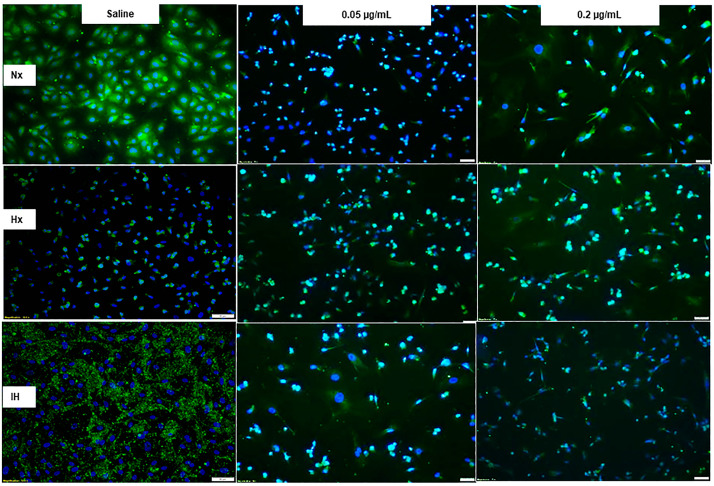
Representative images of the effect of bumetanide on VEGF-A immunoreactivity (green) counterstained with DAPI (blue) in cultured human microvascular retinal endothelial cells (HMRECs) exposed to normoxia (Nx), hyperoxia (Hx, 50% O_2_), or intermittent hypoxia (IH) for 72 h. Images were captured at 20× magnification (scale bar is 50 µm). Quantitative analysis of VEGF immunoreactivity is presented in Table 1.

**Figure 5:**
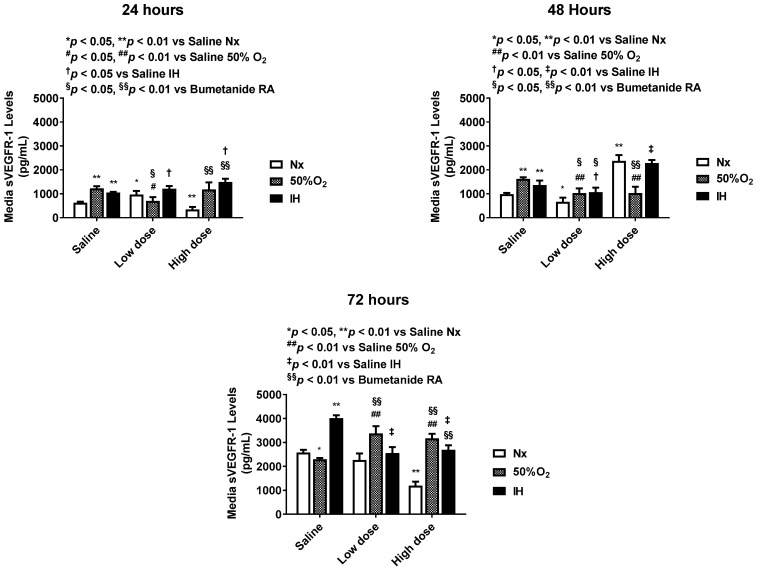
Dose-response effect of bumetanide on soluble VEGF receptor (sVEGFR)-1 levels in the media from cultured human microvascular retinal endothelial cells (HMRECs) exposed to normoxia (white bar), hyperoxia (50% O_2_, shaded bar), or intermittent hypoxia (IH, black bar) for 24, 48, or 72 h. Data were analyzed using two-way ANOVA with Dunnett’s post hoc test. Data are mean ± SEM (8 samples/group). * *p* < 0.05, ** *p* < 0.01 versus saline RA; ^#^ *p* < 0.05, ^##^ *p* < 0.01 versus saline 50% O_2_; ^†^ *p* < 0.05, ^‡^ *p* < 0.01 vs. saline IH; and ^§^ *p* < 0.05, ^§§^ *p* < 0.01 versus bumetanide treatment in RA.

**Figure 6:**
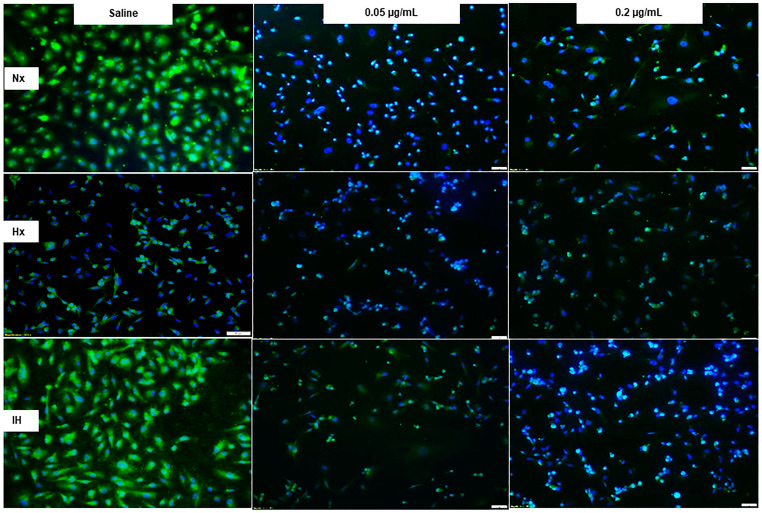
Representative images of the effect of bumetanide on VEGFR-1 immunoreactivity (green) counterstained with DAPI (blue) in cultured human microvascular retinal endothelial cells (HMRECs) exposed to normoxia (Nx), hyperoxia (Hx, 50% O_2_), or intermittent hypoxia (IH) for 72 h. Images were captured at 20× magnification (scale bar is 50 µm). Quantitative analysis of VEGFR-1 immunoreactivity is presented in Table 1.

**Figure 7:**
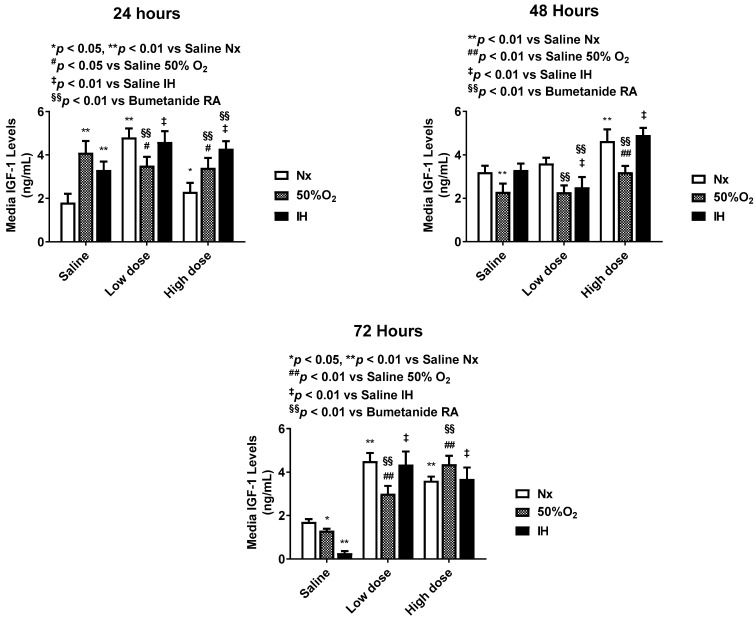
Dose-response effect of bumetanide on insulin-like growth factor (IGF)-1 levels in the media from cultured human microvascular retinal endothelial cells (HMRECs) exposed to normoxia (white bar), hyperoxia (50% O_2_, shaded bar), or intermittent hypoxia (IH, black bar) for 24, 48, or 72 h. Data were analyzed using two-way ANOVA with Dunnett’s post hoc test. Data are mean ± SEM (8 samples/group). * *p* < 0.05, ** *p* < 0.01 versus saline RA; ^#^ *p* < 0.05, ^##^ *p* < 0.01 versus saline 50% O_2_; ^‡^ *p* < 0.01 vs. saline IH; and ^§§^ *p* < 0.01 versus bumetanide treatment in RA.

Bumetanide also significantly decreased media VEGF levels, which progressively increased in the placebo saline group, which is reflective of cell proliferation (Figure 3). This response was reflected in the cells at 72 h (Figure 4), the quantitative analysis of the immunoreactivity in Table 1, and immunoreactivity of HIF_1α_ in the cells at 72 h (Supplemental Figure S1). sVEGFR-1 is an endogenous VEGF inhibitor and splice variant of the membrane VEGFR-1 subtype. sVEGFR-1 is secreted in large amounts by endothelial cells to curtail VEGF levels. In the saline controls, the sVEGFR-1 levels increased significantly with Hx and IH exposure at 24 and 48 h. While the high-dose of bumetanide had an immediate effect, causing elevations in sVEGFR-1 at 24, 48 and 72 h, there was a latent effect in response to the low dose, which resulted in similar elevations at 72 h. Nevertheless, both doses caused elevations in the sVEGFR-1 levels (Figure 5). Interestingly, membrane type VEGFR-1 was robustly expressed in saline-treated cells exposed to IH, while treatment with bumetanide decreased cell size and VEGFR-1 expression (Figure 6, Table 1). VEGFR-2 (Supplemental Figure S2), VEGFR-3 (Supplemental Figure S3), and NP-1 (Supplemental Figure S4) were not similarly affected by bumetanide. IGF-I is a permissive factor for VEGF, and low levels have been shown to be associated with the risk of ROP in preterm infants. In the saline controls, IGF-I levels were higher at 24 and 48 h, but by 72 h, the levels declined in the Hx and IH groups. Bumetanide preserved IGF-I levels in all oxygen conditions and particularly at 72 h (Figure 7). In the cells, bumetanide decreased IGF-I expression with Nx and Hx exposure but increased it during IH exposure, reflecting the measurements in the media (Supplemental Figure S5). IGF-IR immunoreactivity was moderately decreased with the high dose of bumetanide in IH (Supplemental Figure S6).

### 2.4. Effect of Bumetanide on AQP Immunoreactivity

**Figure 8:**
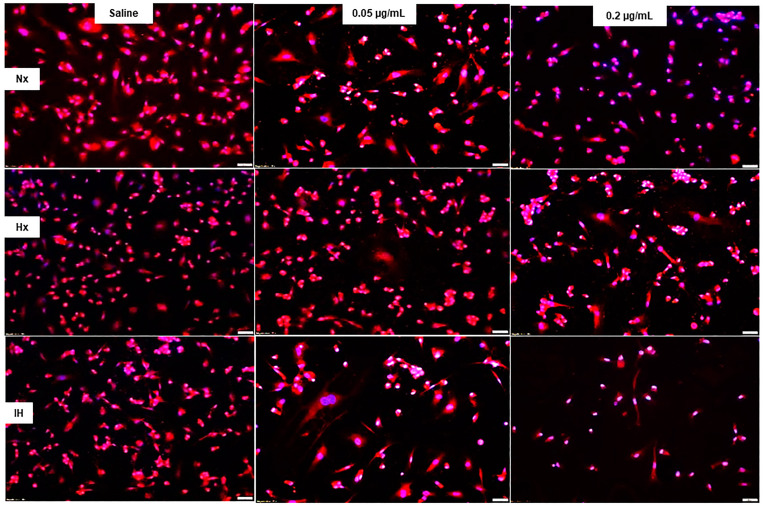
Representative images of the effect of bumetanide on aquaporin (AQP)-1 immunoreactivity (red) counterstained with DAPI (blue) in cultured human microvascular retinal endothelial cells (HMRECs) exposed to normoxia (Nx), hyperoxia (Hx, 50% O_2_), or intermittent hypoxia (IH) for 72 h. Images were captured at 20× magnification (scale bar is 50 µm). Quantitative analysis of AQP-1 immunoreactivity is presented in Table 1.

**Figure 9:**
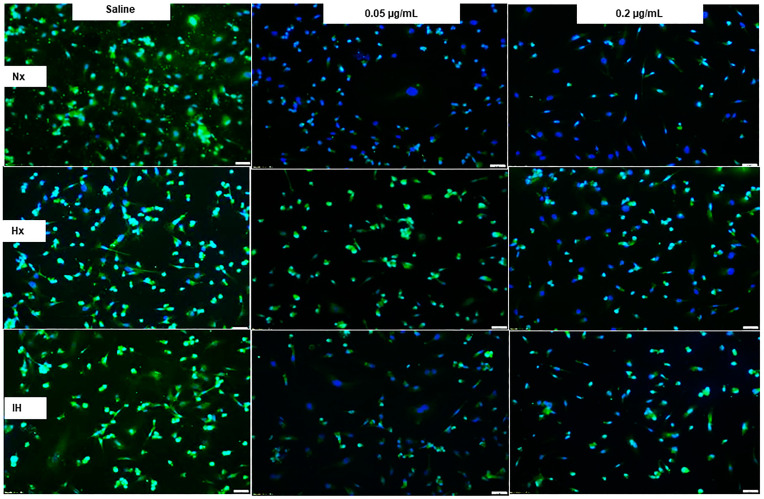
Representative images of the effect of bumetanide on aquaporin (AQP)-4 immunoreactivity (red) counterstained with DAPI (blue) in cultured human microvascular retinal endothelial cells (HMRECs) exposed to normoxia (Nx), hyperoxia (Hx, 50% O_2_), or intermittent hypoxia (IH) for 72 h. Images were captured at 20× magnification (scale bar is 50 µm). Quantitative analysis of AQP-4 immunoreactivity is presented in Table 1.

AQP-1 immunoreactivity (red) in cells at 72 h is presented in Figure 8. Bumetanide had no appreciable effect on AQP-1 immunoreactivity in all oxygen environments, although cell number and size decreased, particularly with the high dose. This is reflected in the quantitative analysis (Table 1). In contrast, AQP-4 immunoreactivity (green) as well as cell number and size were reduced with bumetanide in Hx and IH, (Figure 9, Table 1).

### 2.5. Effects of Bumetanide on Tube Formation Capacity

**Figure 10:**
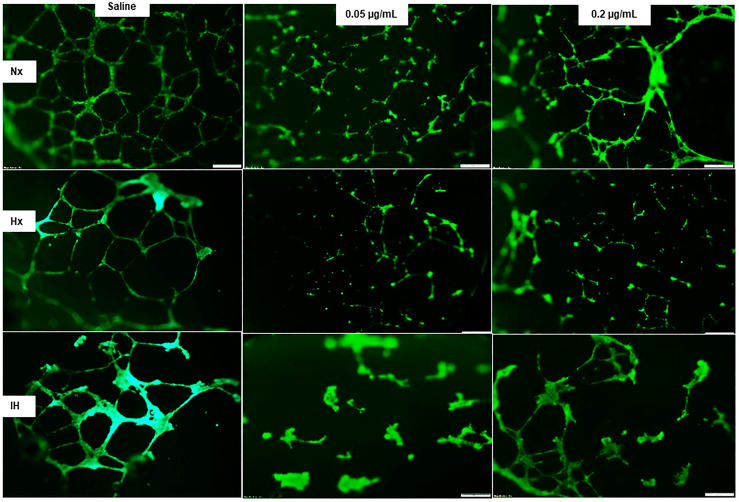
Dose-response effect of bumetanide on tube formation capacity of cultured human microvascular retinal endothelial cells (HMRECs) exposed to exposed to normoxia (Nx), hyperoxia (Hx, 50% O_2_), or intermittent hypoxia (IH) for 72 h. Cells from each group were plated at 2 × 10^5^ in 50 µL media in each well. Cells were labelled with BD calcein AM Fluorescent dye (green). Images were captured at 4× magnification (scale bar is 200 µm). Tube formation assays were conducted in three 96-well plates, one in Nx, one in Hx, and one in IH. In each plate, 32 wells were treated with saline, 32 with low-dose bumetanide (0.05 µg/mL), and 32 with high-dose bumetanide (0.2 µg/mL). Quantitative analysis for the number of tubes formed in each group is presented in Table 1.

Angiogenesis is characterized by a number of cellular events, including endothelial cell migration, invasion, and differentiation into capillaries. We used in vitro tube formation assays as a model to study endothelial cell function and the ability to form tubes in different oxygen environments and the effect of bumetanide on this process. The tube formation capacities in response to bumetanide are presented in Figure 10. Under all of the oxygen conditions, the HMRECs treated with placebo saline formed into networks of branching polygons and an anastomosis of tubes, although reduced tube formation was noted in Hx and IH. In IH, saline controls displayed thicker polygonal cells. Both doses of bumetanide reduced the capacity to form tubes in Nx and reduced this ability to a greater extent in Hx and IH. This is reflected in Table 1.

### 2.6. Quantitative Analysis

Table 1 shows a reduced number of cells with both bumetanide doses in all oxygen conditions. VEGF immunoreactivity declined with both doses in Nx and IH but only with the high dose in Hx. VEGFR-1 also declined with both doses in all oxygen conditions. No change was observed with AQP-1; however, AQP-4 was reduced with both doses in all oxygen conditions. The number of tubes formed decreased with bumetanide in RA and IH and in Hx with the high dose.

### 2.7. Effect of Bumetanide on AQP Gene Expression in HMRECs

Table 2 lists the gene expression of various AQPs in HRECs exposed to Nx, IH, and Hx in response to bumetanide at 72 h post treatment. While both doses significantly downregulated most AQPs, the low dose was more effective in Nx and IH conditions. The most significant finding was the suppressive effect of the low dose on both AQP-1 and AQP-4 in Nx and IH. There was a decrease in the AQP-1 expression with both bumetanide doses, but this difference did not reach statistical significance in any of the oxygen conditions. Both doses of bumetanide decreased the AQP-4 expression in all oxygen conditions, but significance was achieved with the low dose in RA and IH. Both doses decreased the AQP-2 expression, but only the low dose decreased AQP-5 and AQP-6 expression in Nx and IH. AQP-7 expression was decreased with the low dose in Nx and IH and with the high dose in Hx.

## 3. Discussion

The overarching goal of the present study was to determine whether bumetanide is a viable pharmacologic agent for the suppression/prevention of IH-induced oxidative stress and angiogenesis in HMRECs. The major findings of this study are that (1) bumetanide decreases IH-induced oxidative stress but that the effect is not immediate; (2) bumetanide suppresses cell cytotoxicity, an effect that may be due to inhibition of NKCC1, which has been shown to be associated with cytotoxic edema; (3) the anti-angiogenesis effect of bumetanide may involve the suppression VEGF and its receptors; (4) bumetanide induction of sVEGFR-1 also suggests the involvement of the endogenous VEGF trap. However, based on the latent suppressive effect, it is likely that sVEGFR-1 induction occurs as a consequence of decreased VEGF and not directly due to bumetanide action; (5) bumetanide increased IGF-I media levels although the effect was latent. Whether these high levels at 72 h are due to increased secretion by the cells or to decoupling from its binding proteins remain to be determined; (6) bumetanide appears to be a more potent inhibitor of AQP-4 than AQP-1, at least in the setting of neonatal IH; and (7) reduced tube formation capacity with bumetanide likely results from the suppression of VEGF and its receptors. Collectively, these data show for the first time that bumetanide curtails IH-induced angiogenesis, oxidative stress, and cytotoxicity, suggesting a pharmacologic potential for pathologic angiogenesis.

Repeated, brief IH episodes with resolution in either room air or hyperoxia, is frequently experienced by ELGANs. IH is associated with oxidative stress [39] and poor neonatal outcomes [40,41,42,43]. F-isoprostanes are a family of prostaglandin-like compounds that are stable biomarkers of lipid peroxidation originating from oxidation of arachidonic acid and docosahexaenoic acid [44,45], independent of the cyclooxygenase (COX) enzyme [46]. They are potent vasoconstrictors, and their actions are mediated via the thromboxane TP receptor, and the prostaglandin F (PGF)_2α_ FP receptor. The most commonly studied compound is 8-iso-PGF_2α_, also known as 8-epiPGF_2α_ [44,45,46,47]. F2-isoprostanes represent the most accurate and reliable method to assess oxidative stress status in vivo [48]. In our study, treatment with bumetanide in Hx and IH suppressed 8-isoPGF_2α_ at 24 and 72 h, particularly with the high dose. However, treatment in RA resulted in a dose-dependent elevation. The reason for these differences is not clear. Currently, there are no similar studies to confirm or refute our findings. However, we speculate that the effect may be secondary to increased cell survival and angiogenesis at 72 h, an effect that was curtailed with Hx and IH. Studies have shown an association between angiogenesis and ROS is involved in angiogenesis. Further studies are warranted to determine the exact mechanism.

Lactate dehydrogenase (LDH) is a glycolytic enzyme that oxidizes lactate to pyruvate. It is a cytosolic protein that is released upon cell lysis and is associated with apoptosis or necrosis. Due to its high stability and reliability, LDH activity in cell media is commonly used as a direct measurement of cytotoxicity [49,50]. Examination of the % cytotoxicity using the LDH assay showed that exposure to Hx and IH are detrimental to cell survival, with IH being more harmful. Notably, both bumetanide doses equipotently prevented this effect at all time intervals. This finding of bumetanide cytoprotection concurs with previous reports [51] and suggests that even low doses can have beneficial outcomes. Exposure of cells to a hyperoxic environment results in toxicity, the severity of which depends on the degree and duration of hyperoxia [52]. Toxicity resulting from hyperoxia is due to the production of reactive oxygen species (ROS) in excess of ROS scavengers (i.e., antioxidants). These ROS include the superoxide anion, hydrogen peroxide (H_2_O_2_), and lipid peroxides. The excessive production of the superoxide anion and H_2_O_2_ reacting with free iron generates the reactive hydroxyl radical, which is involved in lipid peroxidation. Similarly, IH and reoxygenation result in the production of ROS, leading to oxidative stress [39,53]. Oxidative stress activates chloride cotransporters as well as stress signaling pathways. As such, chloride cotransporters are intrinsic membrane proteins that move Na^+^, K^+^, and Cl^−^ ions across plasma membranes [54]. The bumetanide-sensitive cotransporter NKCC1 is markedly reduced by bumetanide [55]. NKCC1 is also involved in cell proliferation and differentiation [56]. NKCC1 and Na+ overload causes cytotoxic edema, apoptosis, and necrosis [57]. The reduction in oxidative stress observed in our study could be secondary to decreased NKCC1 by bumetanide and subsequently a reduction in cell proliferation.

Angiogenesis involves endothelial cell differentiation, proliferation, and migration, which lead to the formation of vessels [58]. AQPs play a critical role in this process [59]. AQPs are polarized to the leading edge of migrating cells, where they function to drive the water influx facilitating cell migration [60]. Cells overexpressing AQP-1 have accelerated HIF stabilization [61,62,63], and AQP-1 deletion impairs angiogenesis [64]. Bumetanide reduced VEGF in the media and cells as well as decreased the expression of VEGF receptors, which was expected, as previously demonstrated [37,38]. Angiogenesis is regulated by VEGF, a potent EC mitogen and vascular permeability factor that mediates its action via binding to tyrosine kinase receptors (VEGFR-1, VEGFR-2) or the semaphorin receptor (neurophilin-1). VEGF is highly upregulated by hypoxia through the stabilization of HIF_1α_ [65]. The regulation of HIF_1α_ is complex and involves multiple levels of control in nuclear translocation, transcriptional activation, protein degradation, and stabilization [66]. While HIF_1α_ is induced by hypoxia, studies have shown that chronic hyperoxia can induce HIF_1α_ via ROS. During hyperoxia, increased levels of oxygen availability enhance the production ROS, which stabilizes HIF_1α_, resulting in a paradoxical increase. This phenomenon has been shown [67,68] and explained by Jamieson D et al. [52]. The binding of VEGF to VEGFR-2 is thought to mediate the major angiogenic effects of VEGF. In the present study, both VEGF and VEGFR-1 were decreased but not VEGFR-2, VEGFR-3, or NP-1. VEGFR-1 is most strongly expressed in ECs [69]. VEGF binds to VEGFR-1 with a ten-fold higher affinity than it does to VEGFR-2; however, VEGFR-2 is required for endothelial cell proliferation, migration, and survival [70]. Therefore, it seems reasonable that a drastic reduction in VEGF would also be reflected by VEGFR-1 and that the possible mechanism of the inhibitory effect of bumetanide may be the inhibition of HIF_1α_/VEGF.

The VEGFR-1 gene encodes two polypeptides: a transmembrane receptor with a tyrosine kinase domain and an extracellular, soluble form that lacks the tyrosine kinase domain, sVEGFR-1 [71,72]. sVEGF-R1/sFlt-1 is generated by the differential splicing of the flt-1 mRNA [73]. sVEGFR-1 is an endogenous inhibitor of VEGF and decreases the expression of the membrane type VEGFR-1, which promotes angiogenesis. It acts as a VEGF “trap” and prevents the overgrowth and disorganization of ECs, whereas the membrane-bound VEGFR-1 serves to promote vascular growth and development [74]. Studies show that sVEGFR-1/sFlt-1 is involved in guiding vessel sprouting and morphogenesis in developing blood vessels [75,76]. The mechanism involves the inactivation of VEGF action on either side of the sprout, thus providing a corridor for the emerging vessel. In our study, both doses of bumetanide increased sVEGFR-1 secretion by HMRECs in a time-dependent manner, which was correlated with reductions in VEGF and membrane VEGFR-1 expression, confirming its anti-angiogenesis properties and possible therapy applications for proliferative retinopathies. To our knowledge, this is the first report of the bumetanide effects on sVEGFR-1 as well as the first report to suggest that bumetanide induction of sVEGFR-1 could conceivably play a role in decreased angiogenesis. Interestingly, IGF-I was increased with bumetanide. While there are no studies showing a direct link between bumetanide and IGF-I, there are reports of IGF-I induction of sVEGFR-1 in endothelial cells [77], IGF-I suppression of NKCC1 similar to bumetanide [78], and IGF-I reduction of the NKCC1/KCC2 [79]. These studies show synergism among sVEGFR-1, IGF-I, and bumetanide. IGF-I is a potent mitogen, survival, and anti-apoptotic factor for ECs. It is an important mediator of cell growth and differentiation [80] as well as angiogenesis and tube formation [81]. Bumetanide elevation of IGF-I secretion by the HRECs may help to preserve cell survival and to reduce cytotoxicity, regardless of the suppressive effect on VEGF.

In this study, we found that bumetanide suppressed AQP-4 more effectively than AQP-1, and the effect was more robust in normoxia. AQP-1 is strongly expressed in endothelial cells [82,83], where it promotes angiogenesis. Studies show that AQP-1 promotes tumor progression [84], and its blockade curtailed the migration and tube formation of ECs [85]. While we did not see appreciative effects of bumetanide on AQP-4, there were reductions in cell size and numbers in the high-dose groups, particularly in IH. These findings confirm the efficacy of bumetanide for preventing IH-induced angiogenesis. On the other hand, both doses of bumetanide were more suppressive against AQP-4, particularly in RA. AQP-4 is the most abundant AQP in the brain and retina [86], where it plays a role in water homeostasis [87]. In the retina, AQP-4 was preferentially expressed in Müller cells and astrocytes [88,89]. Studies show an association between AQP-4 and VEGF induction [90], confirming its role in angiogenesis. The suppression of AQP-4 prevents the binding of HIF_1α_ to the VEGF promoter, thus reducing hypoxia induced-retinal damage [91]. Our findings agree that bumetanide-induced suppression of AQP-4 results in the subsequent inhibition of HIF_1α_ and VEGF, slowing EC migration.

## 4. Materials and Methods

### 4.1. Cells

HMRECs (ACBRI-181) were purchased from Cell Systems (Kirkland, WA, USA) at 80% confluence (1.5 × 10^6^ cells) and were acclimatized for 2–3 h in an incubator at 37 °C prior to plating in a specialized medium in P75 flasks. Cells were activated with culture boost containing growth factors, antibiotics (Bac-off, Kirkland, WA, USA), and 5% amphotericin B. Cell media was changed every 2 days, and the cells were passaged at 80% confluence. The cells were seeded onto 24-well plates (4 × 10^4^ cells in 0.5 mL media/well) coated with an extracellular matrix product that promotes cell attachment and were incubated at 37 °C and 100% humidity. The number of cells was determined with a TC20 automatic cell counter (BioRad Life Sciences, Hercules, CA, USA). Cell numbers were determined to be similar in each treatment and oxygen group at the start of the experiment.

### 4.2. Experimental Design

Twenty-four well plates were placed in (1) normoxia (Nx) conditions with 5% CO_2_; (2) hyperoxia (Hx, 50% O_2_, 5% CO_2_); or (3) IH (50% O_2_ with brief, 1-min clustered episodes of 5% O_2_; 5% CO_2_). In each oxygen environment, the plates were treated with either (1) low-dose (0.05 µg/mL) or (2) high-dose (0.2 µg/mL) bumetanide diluted in sterile normal saline or (3) equivalent volumes sterile normal saline. Bumetanide sterile injection solution (0.25 mg/mL) was used (Hospira, Inc, Lake Forest, IL, USA). The doses of bumetanide were based on doses used in neonates [33]. On the day of experiment, the media were replaced with fresh media containing drug or placebo saline, and the cells were randomly assigned to the various oxygen environments. Media and cells were harvested at 24, 48, and 72 h post treatment for a total of 27 × 24-well plates. For media samples, three wells from each plate were pooled for a total of eight samples per group. Media samples were analyzed for 8-isoPGF_2α_ (biomarker for oxidative stress), VEGF, soluble VEGF receptor (sVEGFR)-1, insulin-like growth factor (IGF)-I (biomarkers for angiogenesis), and cytotoxicity (LDH assay). For real-time PCR analyses, cells from four wells in each plate were pooled, for a total of four samples per group. Real time PCR assays were conducted using customized AQPs PCR array plates (Qiagen, Germantown, MD, USA). Cells from the remaining two wells per plate were used for the analysis of the tube formation capacity. For the immunoreactivity of AQP, angiogenesis biomarkers, and receptors, separate experiments using cells plated on 16-well culture slides (Fisher Scientific, Pittsburgh, PA, USA) and exposed to similar conditions and treatments were conducted.

### 4.3. Hx and IH Profiles

Cells exposed to Hx and IH were placed into specialized dual sub-chambers (PROOX model 110 oxygen regulator, Biospherix, Redfield, NY, USA) attached to a C42 oxycycler (Biospherix, Parish, NY, USA). The oxycycler supplied O_2_, N_2_, and CO_2_ to the sub-chambers according to the oxygen profile generated to stimulate neonatal IH [92]. The oxygen environment was monitored with oxygen sensors inside the chambers. For the Hx profile, oxygen was set continuously at 50% and remained constant until the end of the experiment. For the IH profile, oxygen was set at 50% for 30 min followed by three 1-min hypoxic (5% O_2_) events, each 10 min apart, for a total of eight clustered episodes/day, consistent with the severe OIR model developed in our laboratory [24,38,93,94,95,96,97,98]. This model simulates neonatal IH experienced by extremely low-gestational age neonates at risk for severe ROP and has consistently resulted in severe OIR in rats. Hypoxia was adjusted for the cells. Instead of the 12% O_2_ used in the animal model, we used 5% O_2_. The oxygen content in the media was continuously monitored using an oxy-validator with an oxygen sensor (BioSpherix) inserted directly into the media of a sacrificial well with cells.

### 4.4. Assay of 8-isoPGF_2α_

Isoprostanes are produced by the non-enzymatic peroxidation of arachidonic acid through ROS. The biomarker 8-isoprostane, or 8-isoPGF_2α_, is commonly studied and is abundantly generated in vivo during oxidative stress and lipid peroxidation, and it is a reliable and proven biomarker for oxidative stress [44,45,46,47]. Levels of 8-isoPGF_2α_ in the media were determined using commercially available enzyme immunoassay kits purchased from Enzo (Ann Arbor, MI, USA), according to the manufacturer’s protocols.

### 4.5. Assay of Cytotoxicity

Lactate dehydrogenase (LDH) is a soluble enzyme that is released in the media due to cell damage or lysis and is a good measure of cytotoxicity. LDH in the media was determined using the cytotoxicity assay kit (Cayman Chemical, Ann Arbor, MI, USA), according to the manufacturer’s protocols. In each sample, % cytotoxicity was determined at absorbance 490 using the formula: [experimental sample LDH minus background LDH divided by maximum release (cells killed with 10% Triton-100) LDH minus background LDH] × 100.

### 4.6. Assay of Angiogenesis Biomarkers

Levels of VEGF, soluble VEGFR-1 (sVEGFR-1), and IGF-I in the media were determined using commercially available human sandwich enzyme immunoassay kits (R&D Systems, Minneapolis, MN, USA), according to the manufacturer’s protocols.

### 4.7. Tube Formation Assay

Becton Dickinson (BD)-BioCoat Angiogenesis System-EC tube formation 96-well plates (BD Biosciences, Bedford, MA, USA) were used. Cells from each group were plated at 2 × 10^5^ in 50 µL media in each well. The plates were incubated for 16–18 h at 37 °C and were then labelled with BD calcein AM Fluorescent dye. The plates were imaged at 4× magnification (scale bar is 200 µm) using an Olympus BX53 microscope, DP72 digital camera, and CellSens imaging software (Olympus, Center Valley, PA, USA). Tube formation assays were conducted in three 96-well plates, one in Nx, one in Hx, and one in IH. In each plate, 32 wells were treated with saline, 32 were treated with low dose bumetanide (0.05 µg/mL), and 32 were treated with high-dose bumetanide (0.2 µg/mL). Quantitative analysis of the number of tubes formed was conducted using the count and measure tool from the CellSens imaging software (Olympus). Only fully formed tubes with complete branching polygons forming a central vacuole were counted. Data are presented in Table 1.

### 4.8. Immunoreactivity Assays

Cells were plated at the same time onto sterile 16-well culture slides (Fisher Scientific, Pittsburgh, PA, USA) and were exposed to similar conditions as those described above for the 24-well plates for 72 h. For each group, the number cells at the start of the experiment was 2 × 10^5^ in each slide. At the end of each experimental time, the slides were washed with warm phosphate-buffered saline (PBS), fixed in 4% paraformaldehyde for 15 min, washed with PBS containing 0.05% tween 20 three times, permeabilized by being incubated with 2 mL 0.1% triton-100 in PBS for 15 min on ice, washed with PBS for 5 min twice, and then incubated with 5% blocking serum in PBS with 0.05% tween 20 and 0.05% sodium azide for 1 h. Following this, the cells were incubated overnight at 4 °C with the following antibodies: AQP-1 (rabbit polyclonal IgG, 1:200), AQP-4 (goat polyclonal IgG, 1:200), HIF_1α_ (rabbit polyclonal IgG, 1:200) VEGF (rabbit polyclonal, 1:200), NP-1 (mouse monoclonal IgG, 1:200), IGF-I (mouse monoclonal IgG, 1:200), and IGF-IRα (mouse monoclonal IgG, 1:200) purchased from Santa Cruz Biotechnology (Dallas, TX, USA); VEGFR-1 (goat polyclonal IgG, 1:200) purchased from R&D Systems (Minneapolis, MN, USA); VEGFR-2 (mouse monoclonal IgG, 1:200) purchased from Millipore Sigma (St. Louis, MO, USA); and VEGFR-3 (rabbit polyclonal IgG, 1:200) purchased from Invitrogen Thermo Fisher (Waltham, MA, USA). The cells were washed with PBS and incubated with Alexa Fluor fluorescent secondary antibodies (1:200) purchased from Life Technologies Thermo Fisher (Waltham, MA, USA) overnight. After being washed with PBS/tween 20 twice, the cells were counterstained with DAPI. One drop of prolong antifade was added prior to coverslipping. Cells were imaged at 20× magnification (scale bar is 50 µm) using an Olympus IX73 inverted microscope system and CellSens imaging software (Olympus, Center Valley, PA, USA).

### 4.9. Real Time PCR

Total RNA was harvested from cells by the addition of RNAPro solution (MP Biomedicals, Solon, OH, USA) to the wells. RNA was extracted using the FastPrep-24 system (MP Biomedicals) and was purified using the RNEasy mini cleanup kits (Qiagen, Germantown, MD, USA). Reverse transcriptase was performed using a RT^2^ First Strand kit (Qiagen). Real time PCR assays of AQP expression were conducted (*n* = 4/group) using customized PCR Arrays plates (Qiagen) with a BioRad IQ5 real-time instrument (BioRad, Hercules, CA, USA), as previously described [98].

### 4.10. Statistical Analysis

To determine the differences among the Nx, Hx, and IH oxygen groups and the differences among the treatment groups, two-way ANOVA was used for normally distributed data and the Kruskal–Wallis test was used for non-normally distributed data following Barlett’s test for normality. Post hoc analysis was performed using Dunnett’s test. Significance was set at *p* < 0.05, and data are reported as mean ± SEM. All analyses were two tailed and were performed using SPSS software version 26.0 (SPSS Inc., Chicago, IL, USA) and GraphPad Prism software version 7.0 (GraphPad Inc., San Diego, CA, USA).

## 5. Conclusions

In the setting of IH, bumetanide effectively attenuates the EC tube-formation capacity and decreases angiogenesis. Since bumetanide is a potent inhibitor of AQP-4 and NKCC1, the mechanism may involve either the upregulation of sVEGFR-1, the endogenous VEGF “trap”, and/or inhibition of AQP-4 and NKCC1, both of which are involved in cytotoxic edema and cell death in oxidative stress conditions. While our results provide new insights into some of the valuable associations regarding the anti-angiogenic and antioxidant effects of bumetanide and its pharmacologic potential for reducing pathologic angiogenesis, further dose-finding studies are needed to determine the lowest dose with the maximum therapeutic efficacy and safety.

## Figures and Tables

**Table 1:** Quantitative Analysis.

	Normoxia (Nx)			Hyperoxia (Hx, 50% O_2_)			Intermittent Hypoxia (IH)		
	Saline	Low Dose	High Dose	Saline	Low Dose	High Dose	Saline	Low Dose	High Dose
No. Cells	537.6 ± 105.1	208.4 ± 17.3 **	138.5 ± 15.0 **	289.6 ± 25.8	217.5 ± 11.1 **^,#^	149.8 ± 14.8 **	477.5 ± 32.8	331.0 ± 58.5 ^#^	240.0 ± 26.0 **^,##^
VEGF	949.0 ± 174.4	306.0 ± 24.0 **	254.0 ± 29.1 **	419.8 ± 69.8 #	398.5 ± 20.1	240.5 ± 18.4 *	1132.0 ± 178.4	367.8 ± 68.9 **	260.0 ± 29.8 **
VEGFR-1	504.3 ± 36.1	345.8 ± 46.6 *	368.5 ± 33.0 *	617.3 ± 113.7	158.3 ± 14.3 **^,#^	234.8 ± 47.0 **	1196.8 ± 127.4 ^##^	447.8 ± 68.0 **	583.5 ± 72.7 **^,#^
AQP-1	560.8 ± 76.5	470.7 ± 111	318.8 ± 105.5	550.9 ± 96.6	583.0 ± 45.1	407.3 ± 58.3	504.2 ± 110.3	432.0 ± 67.1	388.5 ± 53.2
AQP-4	1236.6 ± 110	388.6 ± 90.9 **	277.3 ± 52.7 **	1153.0 ± 118	673.0 ± 108 *	771.8 ± 89.7 *^,##^	1623.8 ± 111.1	482.5 ± 66.4 **	1021.5 ± 74.6 **^,##^
No. Tubes	90.3 ± 12.5	38.3 ± 8.9 **	44.9 ± 15.6 *	29.6 ± 8.0 ^##^	16.6 ± 1.0 ^##^	10.2 ± 2.0 **^,#^	45.5 ± 11.0 ^##^	0 ± 0 **^,##^	6.6 ± 2.0 **^,##^

Low-dose bumetanide (0.05 µg/mL); high-dose bumetanide (0.2 µg/mL). VEGF, VEGFR-1, AQP-1, and AQP-4 immunoreactivities correspond to Figure 4, Figure 6, Figure 8 and Figure 9, respectively. The number of tubes formed corresponds to Figure 10. Data were analyzed using two-way ANOVA with Dunnett’s post hoc test. * *p* < 0.05, ** *p* < 0.01 vs. saline; ^#^ *p* < 0.05, ^##^ *p* < 0.01 vs. normoxia (Nx). Data are mean ± SEM (6 measurements/group for immunofluorescence images and no. cells as well as 32 measurements/group for number of tubes).

**Table 2:** Expression of aquaporin (AQP) genes in human retinal endothelial cells exposed to hyperoxia (Hx), intermittent hypoxia (IH), or normoxia (Nx). Data are fold changes from the saline-treated Nx controls.

Genes	Saline Hx	Saline IH	Low Dose Nx	Low Dose Hx	Low Dose IH	High Dose Nx	High Dose Hx	High Dose IH
AQP-1	−1.5 ± 0.09	4.3 ± 0.3	−72.5 ± 17.4 **	1.5 ± 0.2	−41.1 ± 1.8 **	−4.5 ± 3.9	−3.8 ± 2.5	−4.5 ± 1.2
AQP-2	−2.0 ± 4.2	−0.2 ± 1.7	−15.2 ± 2.7 **	−11.7 ± 0.9 **	−12.3 ± 1.8 **	−10.0 ± 0.9 **	−10.6 ± 0.9 **	−16.1 ± 1.5 **
AQP-3	1.4 ± 4.2	2.6 ± 1.7	−4.9 ± 1.3	1.1 ± 0.13	−2.7 ± 1.2	0.01 ± 1.8	−3.4 ± 0.5	−3.2 ± 0.3
AQP-4	1.7 ± 0.9	1.0 ± 2.9	−249.1 ± 95 **	−0.3 ± 3.1	−152.1 ± 19.3 **	−6.1 ± 0.7	−2.5 ± 0.01	−1.7 ± 1.0
AQP-5	−0.3 ± 2.3	13.2 ± 2.5	−243.0 ± 86 **	−8.5 ± 1.7 *	−131.0 ± 10.2 *	−24.5 ± 10.2 **	−6.8 ± 2.8	−35.9 ± 14.6 **
AQP-6	3.6 ± 1.4	−6.9 ± 0.91	−260.3 ± 71.1 **	−24.2 ± 8.8 **	−215.4 ± 34.6 **	−423.2 ± 18.7 **	−18.2 ± 1.6 **	−245.4 ± 65.6 **
AQP-7	1.2 ± 0.3	9.2 ± 1.1 **	−50.7 ± 1.3 **	3.0 ± 0.03	−31.6 ± 2.6 **	−2.0 ± 1.6	−11.2 ± 0.3 **	−1.2 ± 0.3

AQP (aquaporin); Hx (hyperoxia, 50% O_2_); IH (intermittent hypoxia); low-dose bumetanide (0.05 µg/mL); high-dose bumetanide (0.2 µg/mL); hyperoxia (50% O_2_); ** *p* < 0.01 vs. saline Nx (n = four samples per group). Data were analyzed using one-way ANOVA with Dunnett’s post hoc test. Data are mean ± SEM (* *p* < 0.05, ** *p* < 0.01 vs. saline Nx). The minus sign represents downregulation.

## Data Availability

Data is contained within the article and Supplementary Material.

## Supplementary Materials

The following are available online at https://www.mdpi.com/article/10.3390/ph14100967/s1, Figure S1: HIF_1α_, Figure S2: VEGFR-2, Figure S3: VEGFR-3, Figure S4: NP-1, Figure S5: IGF-I, and Figure S6: IGF-IRα.
