# Supplementary material for: Dose Response of Bumetanide on Aquaporins and Angiogenesis Biomarkers in Human Retinal Endothelial Cells Exposed to Intermittent Hypoxia
Source: Pharmaceuticals (Basel). 2021 Sep 24;14(10):967. doi: 10.3390/ph14100967 (PMC8538009; doi:10.3390/ph14100967)
Supplement: Supplementary file 1 [file pharmaceuticals-14-00967-s001.zip › Guzel S_Pharmaceuticals_Supplementary Figures.pptx]

## Slide 1
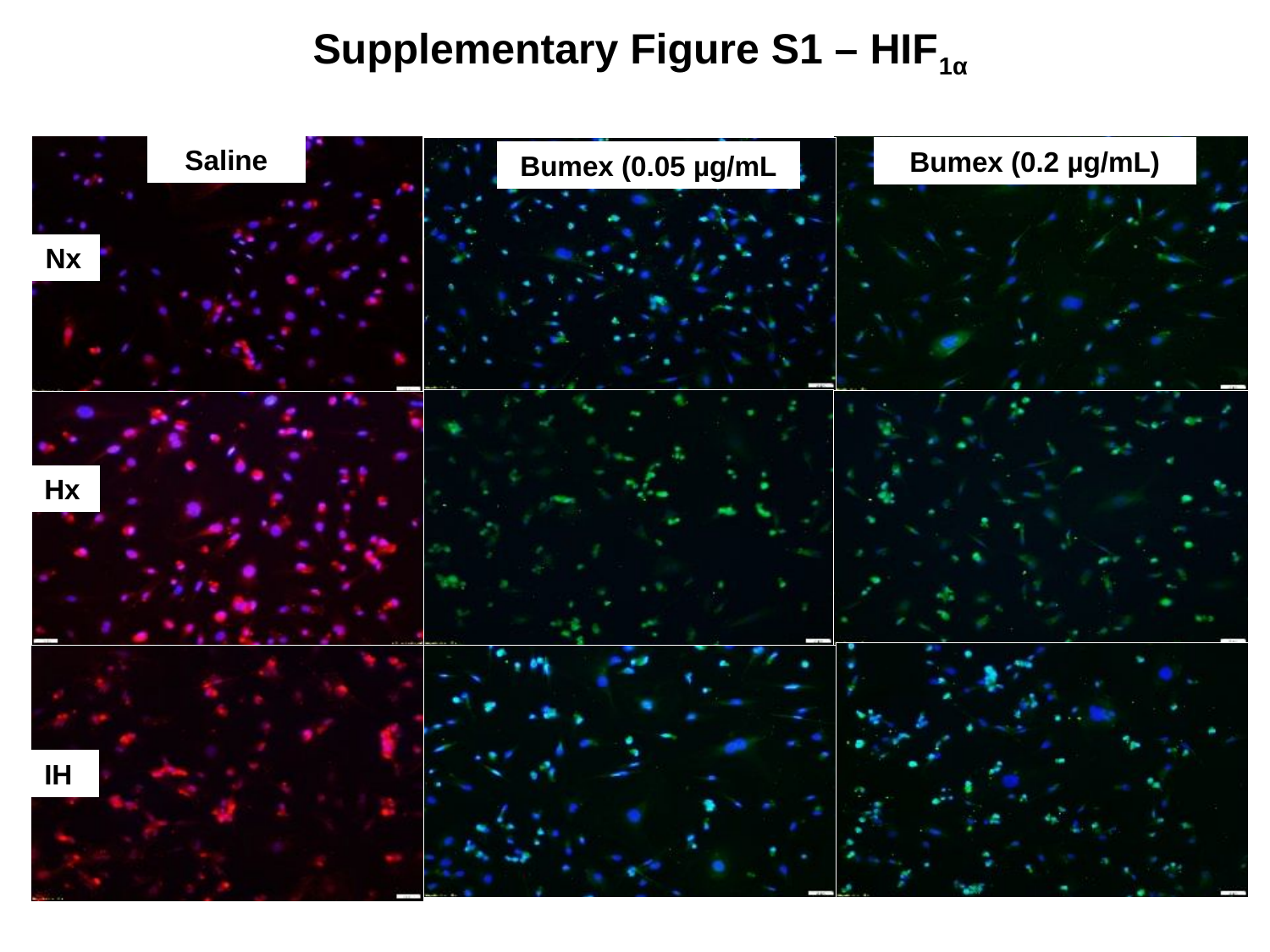

Supplementary Figure S1 – HIF1α
Saline
Bumex (0.2 µg/mL)
Bumex (0.05 µg/mL
Nx
Hx
IH

## Slide 2
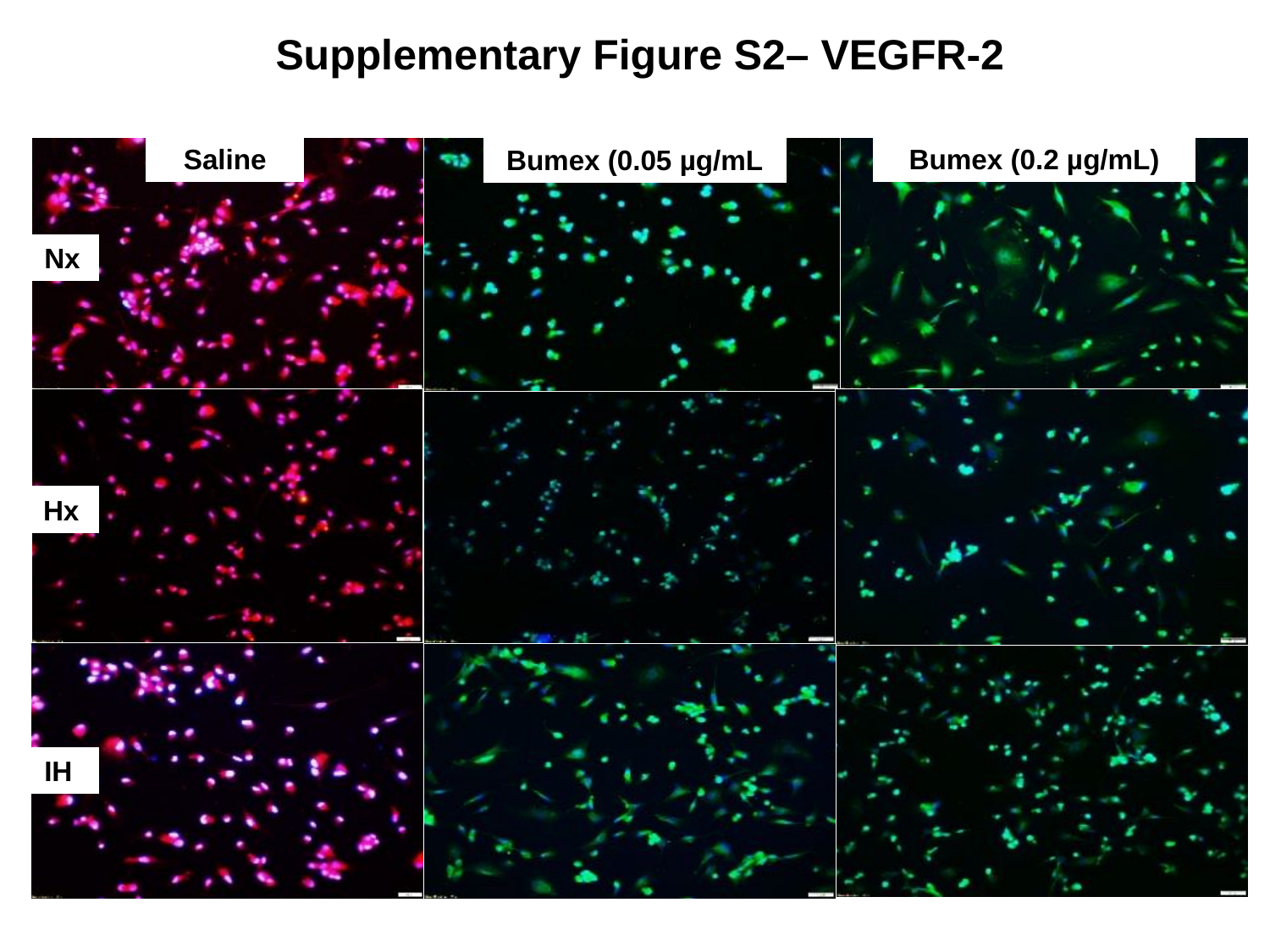

Supplementary Figure S2– VEGFR-2
Saline
Bumex (0.2 µg/mL)
Bumex (0.05 µg/mL
Nx
Hx
IH

## Slide 3
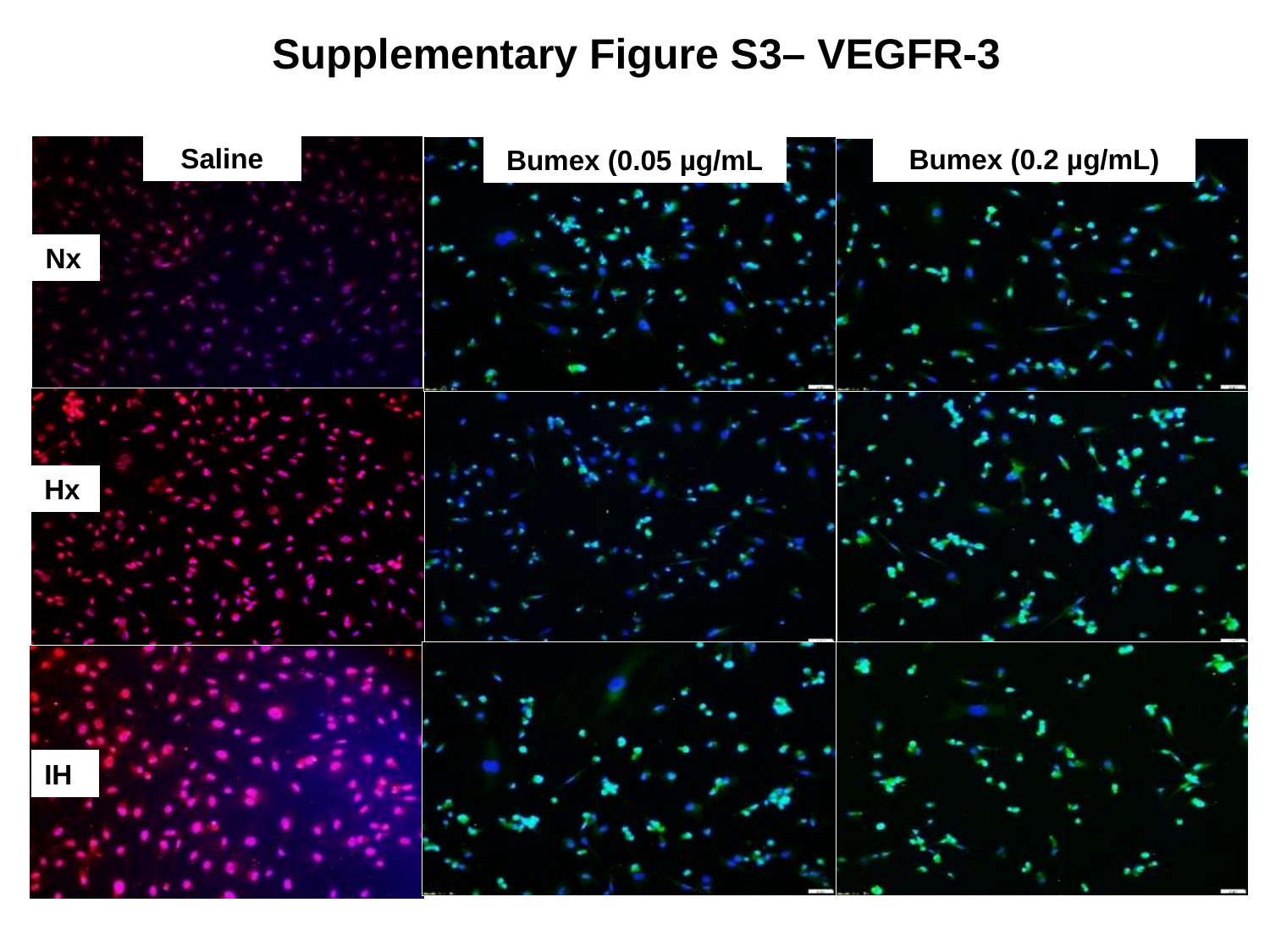

Supplementary Figure S3– VEGFR-3
Saline
Bumex (0.2 µg/mL)
Bumex (0.05 µg/mL
Nx
Hx
IH

## Slide 4
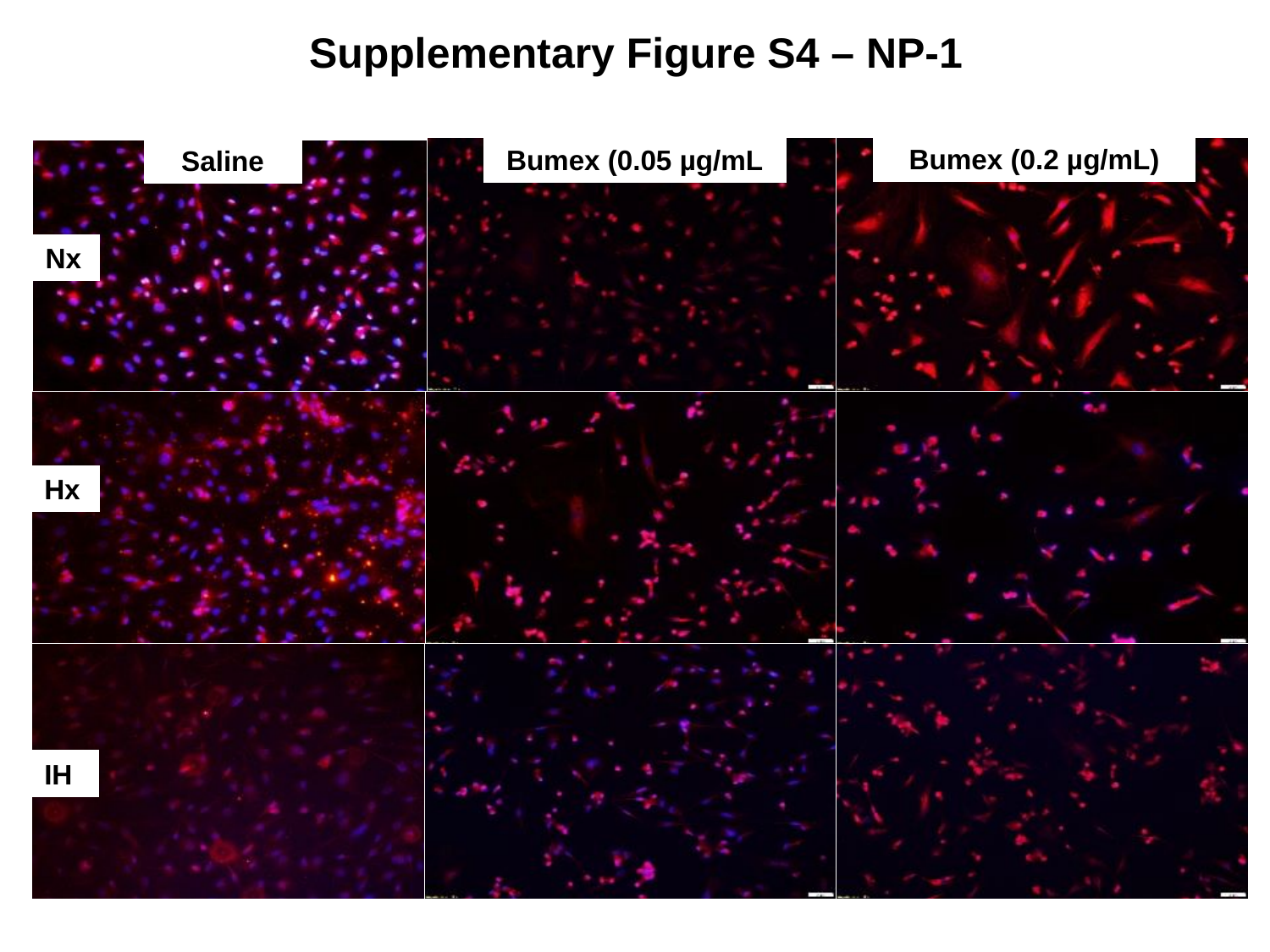

Supplementary Figure S4 – NP-1
Bumex (0.2 µg/mL)
Bumex (0.05 µg/mL
Saline
Nx
Hx
IH

## Slide 5
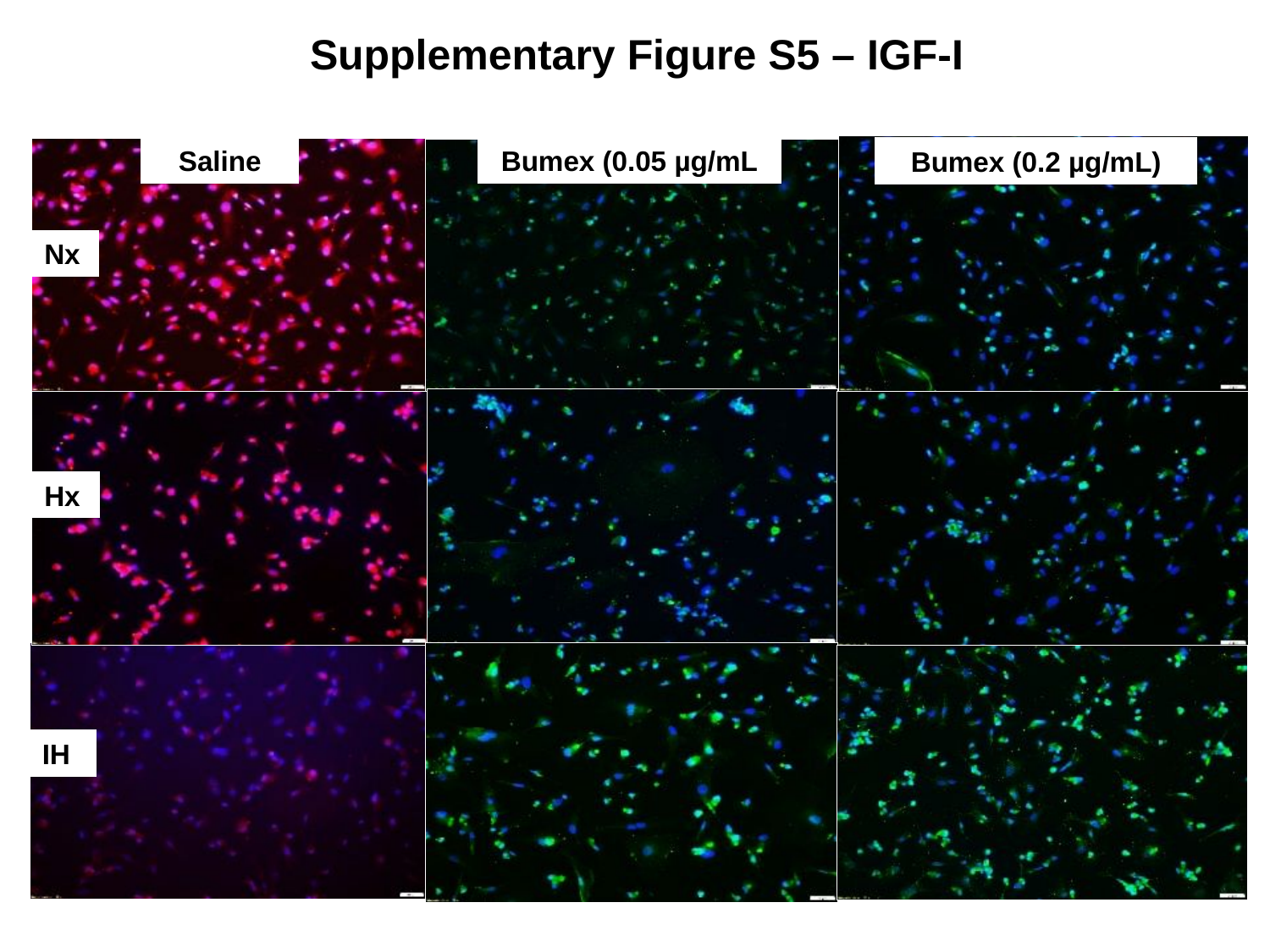

Supplementary Figure S5 – IGF-I
Saline
Bumex (0.05 µg/mL
Bumex (0.2 µg/mL)
Nx
Hx
IH

## Slide 6
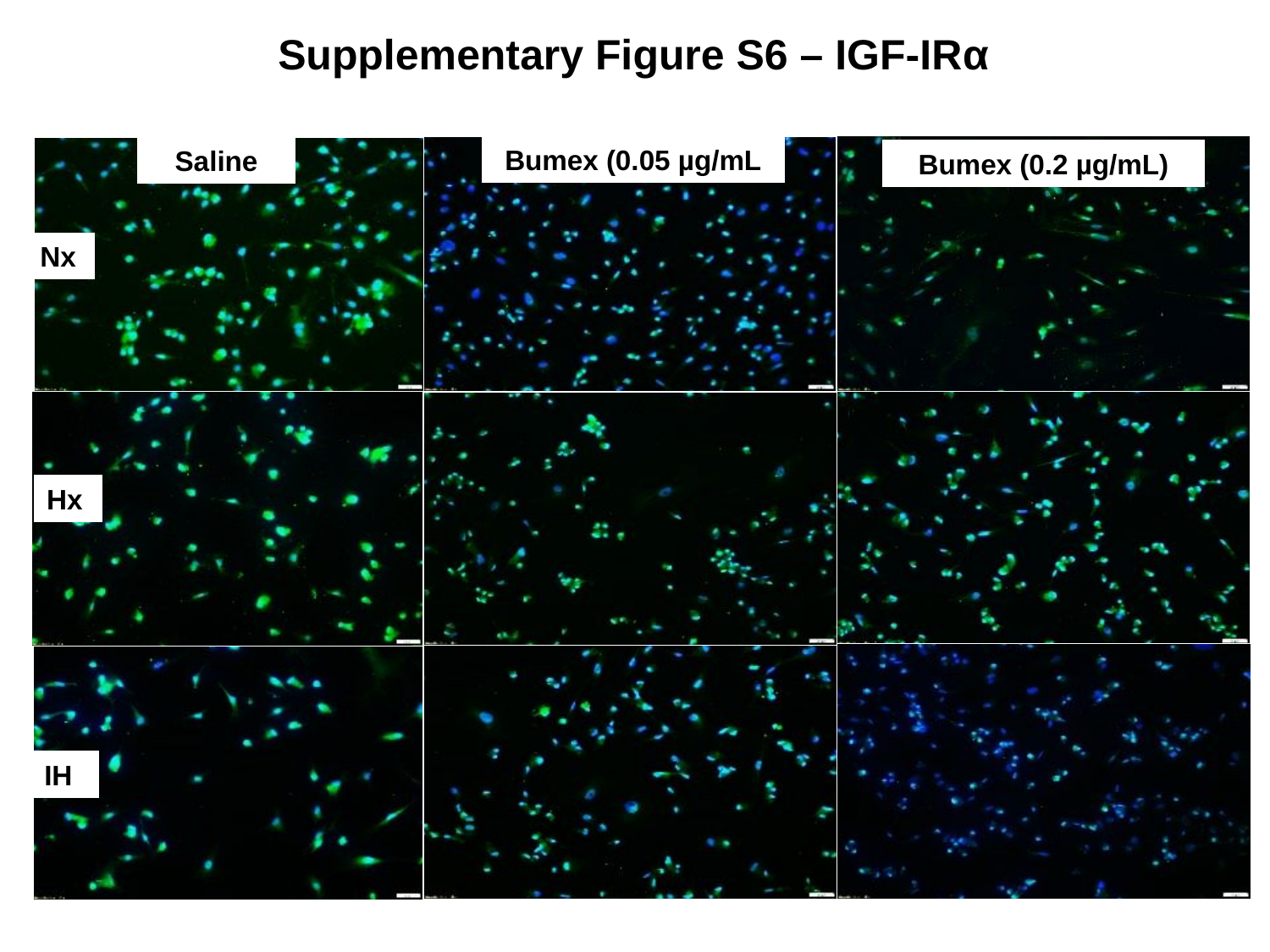

Supplementary Figure S6 – IGF-IRα
Bumex (0.05 µg/mL
Saline
Bumex (0.2 µg/mL)
Nx
Hx
IH
